# Supplementary material for: Patterns of Intron Gain and Loss in Fungi
Source: PLoS Biol. 2004 Nov 30;2(12):e422. doi: 10.1371/journal.pbio.0020422 (PMC532390; doi:10.1371/journal.pbio.0020422)
Supplement: Table S1 — Also available at http://genes.mit.edu/NielsenEtAl/. (4.3 MB ZIP). [file pbio.0020422.st001.zip › NielsenEtAl/html/761.html]

AN8702.1.NCU02113.1.MG02568.1.FG08558.1


```
 CLUSTAL W (1.82) Multiple Sequence Alignments - Introns Inserted


Sequence 1: NCU02113.1	151 aa
Sequence 2: MG02568.1	150 aa
Sequence 3: FG08558.1	149 aa
Sequence 4: AN8702.1	144 aa
Alignment Length: 152 aa
Number Identitical Residues: 108 aa
Alignment Score (without introns) 4922


MG02568.1 	MA-LPKRIVKETERLMAEP2VPGISAVPHEDNLRYFDVEIHGPSQSPYEG1GVFKLDLFL
NCU02113.1	MA-LPKRIVKETERLMAEP2VPGISAVPHEDNLRYFDVEIHGPSQSPYEG1GVFKLELFL
FG08558.1 	MA-LPKRIIKETERLMAEP2VPGISAIPHEDNLRYFDVQIHGPAQSPYEG1GVFNLELFL
AN8702.1  	MAGLPKRIVKETERLLSDP2VPGINAVPHDDNLRYFDVSIHGPTSSPYEG~GIFRLELFL
          	**.*****:******:::* ****.*:**:********.****:.***** *:*.*:***

MG02568.1 	PDDYPMTPPKIRFVTKIYHPNVDKLGRIC~LDVLKN1NWSPALQIRTILLSIQALLGAPN
NCU02113.1	PDDYPMTPPKIRFLTKIYHPNVDKLGRIC~LDVLKN1NWSPALQIRTILLSIQALLGAPN
FG08558.1 	PDDYPMTPPKIRFLTKIFHPNVDKLGRIC~LDVLKN1NWSPALQIRTILLSIQALLGAPN
AN8702.1  	PEDYPMVPPKIRFLTKIYHPNIDRLGRIC1------~NWSPALQIRTILLSIQALLGAPN
          	*:****.******:***:***:*:*****        ***********************

MG02568.1 	PDDPLAPDVAKAWKEDEPAAIKTARDWTLQYATPK-
NCU02113.1	PDDPLAPDVAKTWKENPSVAVATAREWTQQYALPSQ
FG08558.1 	PDDPLAADVAKSWKEDEQAAIATAKEWTAQYAKP--
AN8702.1  	PDDPLANDVAQRWKEDEAAAIKTAKEWTITHAMT--
          	****** ***: ***:  .*: **::**  :* .
```
